# A Review of Magnetic Nanoparticle-Based Surface-Enhanced Raman Scattering Substrates for Bioanalysis: Morphology, Function and Detection Application

**DOI:** 10.3390/bios13010030

**Published:** 2022-12-27

**Authors:** Hanbing Huang, Zhuomin Zhang, Gongke Li

**Affiliations:** School of Chemistry, Sun Yat-sen University, Guangzhou 510006, China

**Keywords:** magnetic nanoparticle-based substrate, surface-enhanced Raman scattering, bioanalysis

## Abstract

Surface-enhanced Raman scattering (SERS) is a kind of popular non-destructive and water-free interference analytical technology with fast response, excellent sensitivity and specificity to trace biotargets in biological samples. Recently, many researches have focused on the preparation of various magnetic nanoparticle-based SERS substrates for developing efficient bioanalytical methods, which greatly improved the selectivity and accuracy of the proposed SERS bioassays. There has been a rapid increase in the number of reports about magnetic SERS substrates in the past decade, and the number of related papers and citations have exceeded 500 and 2000, respectively. Moreover, most of the papers published since 2009 have been dedicated to analytical applications. In the paper, the recent advances in magnetic nanoparticle-based SERS substrates for bioanalysis were reviewed in detail based on their various morphologies, such as magnetic core–shell nanoparticles, magnetic core–satellite nanoparticles and non-spherical magnetic nanoparticles and their different functions, such as separation and enrichment, recognition and SERS tags. Moreover, the typical application progress on magnetic nanoparticle-based SERS substrates for bioanalysis of amino acids and protein, DNA and RNA sequences, cancer cells and related tumor biomarkers, etc., was summarized and introduced. Finally, the future trends and prospective for SERS bioanalysis by magnetic nanoparticle-based substrates were proposed based on the systematical study of typical and latest references. It is expected that this review would provide useful information and clues for the researchers with interest in SERS bioanalysis.

## 1. Introduction

Recently, with the rapid development of biological science and biomedical science, fast and sensitive analysis of typical biotargets as important biomarkers have attracted much more interest due to their important physiological functions associated with biological activities and many diseases [1,2,3,4,5]. The precise analysis of these biotargets would provide the key criteria, which is essential to human health management, clinical diagnosis, food and environmental safety monitoring, etc. Various spectroscopy [6,7,8,9] and chromatography methods involving chromatography-mass hyphenated methods [10,11,12] have been widely applied for bioanalysis. However, since these methods usually require the use of large-size instruments with complicated sample preparation procedures, they cannot meet the growing need for fast detection and point-of-care testing (POCT).

Biosensing methods, such as photoluminescence [13], chemiluminescence [14], surface plasmon resonance (SPR) [15] and surface-enhanced Raman scattering (SERS) sensing [16,17,18], have been well developed in recent years. Compared to traditional techniques, theses optical biosensing methods usually possess fast response and excellent sensitivity and specificity. In particular, SERS is known as an ultrasensitive vibration spectroscopy that can provide unique ‘fingerprint information’ for each molecule and achieve the fast identification of substance composition [19,20,21,22]. SERS has been attractive for bioanalysis due to its rapid and non-destructive analysis process and water-free interference [23,24,25,26,27]. Therefore, SERS has been widely used in the bioanalysis of typical biotargets, such as amino acids [28], proteins [29], nucleotides [30,31] and tumor biomarkers [32]. However, once these biotargets are analyzed in body fluids and living cells, the analysis performance of SERS significantly decreases due to the complexity of biological sample matrices [33,34]. The progress on the rapid development of efficient SERS substrates [35,36] has brought about the dramatically increasing sensing sensitivity of SERS, but the primary challenge of SERS technology is directly related to limited signal reproducibility and analytical precision during biological samples analysis with consideration of the non-selective enhancement and matrix effect. Therefore, it is indispensable to use the suitable separation and enrichment function for SERS substrates to improve the analytical precision and further analytical accuracy for the consequent bioassays.

It has aroused great attention to achieve the promising timeliness of biosensing while maintaining the effective separation and enrichment of targets from complex biological samples via the preparation of efficient and selective SERS substrates. Recently, magnetic nanoparticles (MNPs) with excellent plasmonic property have been introduced to construct magnetic SERS substrates, exhibiting great potential to achieve separation, enrichment and SERS detection all-in-one for bioanalysis as follows. (1) High sensitivity: The plasmonic properties of MNPs can be easily and dramatically tuned to obtain an ideal enhancement factor (EF). Moreover, the generation of more hot spots always accompanies the magnetic trap effect of the MNPs in the separation process, which can further improve the sensitivity. In addition, biological samples exhibit virtually no magnetic background, which benefits the highly sensitive measurements [37]. (2) Excellent selectivity: The good biocompatibility enables the immobilization of biorecognition materials on the surface of MNPs, which can result in more adsorption interactions such as antigen–antibody-specific interaction [38], π–π stacking [39], electrostatic interaction [40], hydrogen bonding [41], etc., to improve analytical selectivity. (3) Satisfied accuracy: Introducing MNP substrates with great separation and enrichment capability for the development of SERS bioassays could reduce the matrix interference and improve the analytical accuracy. (4) Improved efficiency: Separation-based functionalized MNP substrates can be simply and rapidly realized by magnetism to ensure the high efficiency of SERS detection.

**Figure 1:**
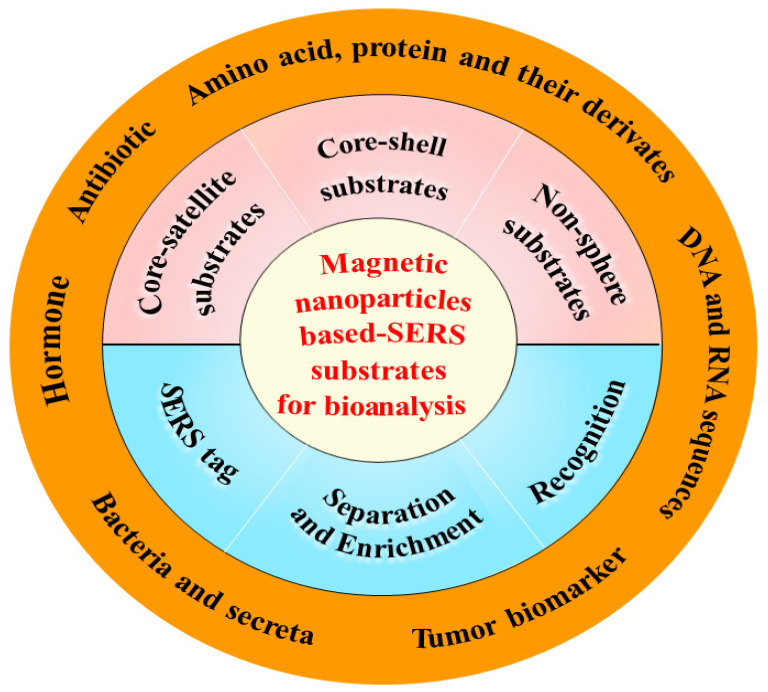
Schemic of magnetic nanoparticle-based SERS substrates for analysis of typical biotargets.

Several works have reviewed the applications of MNPs in SERS technology in the past decade [42,43,44]. These literatures mainly focused on the synthesis methods and applications of MNPs, and the construction strategy of the magnetic SERS method. It is essential to systematically review MNP-based SERS substrates with different morphologies, their functions and applications in SERS identification and accurate quantification of biotargets. This review attempts to present an overview of the recent progress in MNP-based SERS substrates from the aspects of the fabrication and properties of MNP-based substrates with different morphologies and functions for the development of sensitive and accurate SERS bioanalytical methods and their closely related applications for the analysis of typical biotargets (Figure 1). Finally, the challenges and future perspectives of MNP-based SERS substrates for biological analysis were presented.

## 2. Morphologies of Magnetic Nanoparticle-Based SERS Substrates

**Figure 2:**
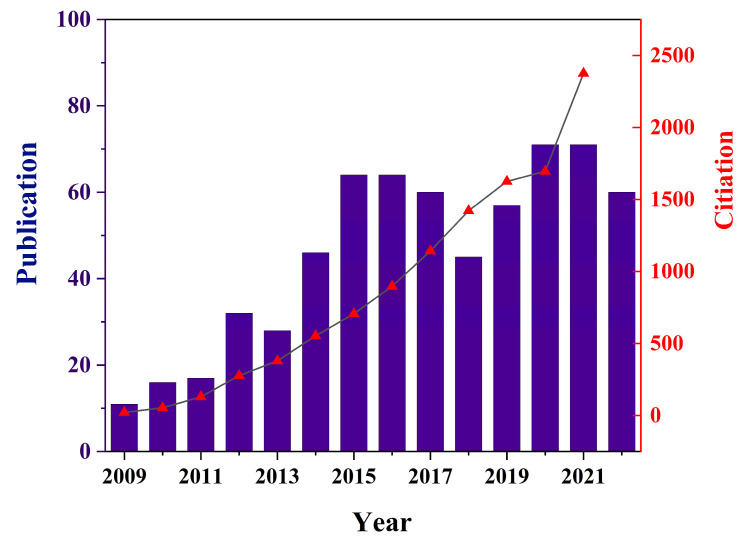
The trends of publication and citation with the keywords of magnetic SERS substrate. The data collected were from 2009 to Nov. 2022 according to *Web of Science*.

Plasmonic MNPs have been studied extensively and been regarded as the ideal SERS substrates due to their incomparable superiorities in SERS analysis of complex samples. The publication and citation trends closely related with magnetic SERS substrates demonstrated the growing trends in recent years, as shown in Figure 2. The usage of MNP-based substrates for bioanalysis can achieve separation, enrichment and SERS detection all-in-one to significantly improve the efficiency of the whole analysis process. Generally, most of the reported MNP-based SERS substrates can be classified as plasmonic shell-coated magnetic core structures like magnetic metal–core–shell nanoparticles (NPs) and magnetic composites loaded with SERS-active NPs. Conventional noble metal nanomaterials, such as Au and Ag NPs, have often been used as plasmonic functional parts of the magnetic substrates to generate localized surface plasmon resonance (LSPR) for the electromagnetic (EM) enhancement of biotargets. The uniformity of the substrates is a key factor for the reproducibility of the Raman signal during SERS analysis, while the SERS activity of the magnetic substrates is closely related to their morphologies. For example, magnetic metal–core–shell NPs, the branched structures of outer-shell plasmonic materials, can provide a high number of hot spots and achieve the excellent enhancement of the Raman signal compared to substrates with a smooth and spherical morphology. Core-satellites magnetic substrates usually exhibit outstanding SERS performance due to the assembled noble metal (e.g., Au, Ag) satellites that can create sufficient hot spots. In this part, the recent progress on the fabrication and properties of MNP-based SERS substrates with different morphologies for bioanalysis will be summarized and reviewed in detail.

### 2.1. Magnetic Core–Shell Nanoparticles

**Figure 3:**
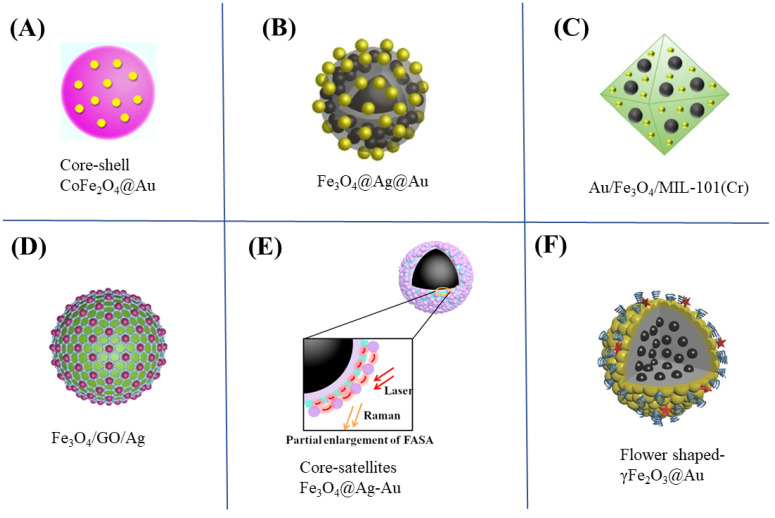
Typical MNP-based SERS substrates with different shapes: (**A**) sphere-like core–shell Fe_3_O_4_@Au. Reproduced with permission from Ref. [46]. Copyright 2016, American Chemical Society. (**B**) Fe_3_O_4_@Ag@Au Reproduced with permission from Ref. [47]. Copyright 2022, Springer. (**C**) Au/ Fe_3_O_4_/MIL-101(Cr) Reproduced with permission from Ref. [48], Copyright 2022, Elsevier. (**D**) Fe_3_O_4_/GO/Ag Reproduced with permission from Ref. [49], Copyright 2022, Elsevier. (**E**) Core–satellites Fe_3_O_4_@Ag–Au Reproduced with permission from Ref. [50], Copyright 2017, Elsevier. (**F**) Flower-shaped γFe_2_O_3_@Au Reproduced with permission from Ref. [51]. Copyright 2017, Wiley.

Plasmonic core–shell NPs have been attractive due to their precisely tunable physiochemical features. Magnetic core–shell NPs, consisting of a magnetic core and outer shell with plasmonic property, have been extensively used as a SERS substrate and exhibit great performance. The SERS activity of core–shell NPs can be easily tuned by regulating the thickness, morphology and shape of a shell, as well as changing the size and shape of the core. In SERS-based bioanalysis, iron oxides (Fe_3_O_4_ and Fe_2_O_3_) are the most frequently used magnetic materials due to their low toxic effect. Meanwhile, the coated shell made of different materials can protect bare MNPs from uncontrollable aggregation and improve their stability in biological samples. Particularly, gold shell has good compatibility for the modification of biorecognition molecules, so Au-coated MNPs have been considered suitable substrates for bioseparation and subsequent SERS analysis. Zhang et al. [45] reported a SERS method to detect a cancer-related microRNA biomarker of miR-141 using Au-coated paramagnetic NPs (Fe_3_O_4_@ Au) as a magnetic substrate and labeled AuNPs as a SERS tag. The hydraulic Fe_3_O_4_@Au MNPs were conjugated with oligonucleotide probes to efficiently capture miR-141 target sequences through hybridization-dependent recognition. The combination of Fe_3_O_4_@Au and AuNPs led to high capture efficiency for microRNA with excellent SERS sensitivity and a limit of detection that was as low as 100 fM. Similarly, He et al. [46] prepared CoFe_2_O_4_@AuNPs and used it as a SERS substrate (Figure 3A) and capture probe for SERS application. The proposed SERS platform achieved a large dynamic range (1 fg/mL~1 ng/mL) and a low detection limit (0.75 fg/mL) for N-terminal pro-brain natriuretic peptide due to the excellent SERS sensitivity and high affinity to the analyte of the magnetic CoFe_2_O_4_@AuNPs substrate.

To improve the dispersion and chemical stability of bare MNPs, a hydrophilic macromolecule, such as polyethyleneimine (PEI), is usually employed as an interlayer between magnetic core and outer shell. Liu et al. [52] combined the SERS technique and lateral flow assay to achieve the simultaneous detection of multiple biomarkers using the magnetic substrate of Fe_3_O_4_@PEI@Au. In the PEI-mediated seed growth strategy, Au NPs were used as seeds to grow an Au shell on the surface of Fe_3_O_4_@PEI by chemical reduction. The PEI interlayer ensured the long-term stability and reproducible SERS effect of Fe_3_O_4_@PEI@Au, which facilitated the quantitative analysis of C-reactive protein (CRP). The Fe_3_O_4_@PEI@Au could be applied to minimally detect 0.01 ng/mL CRP in blood samples without pretreatment. These seed-mediated strategies are also applicable to synthesize Ag-coated MNPs with remarkable SERS performance. SiO_2_ is usually employed as the encapsulation of a magnetic core to ensure the good dispersion and long-term stability of Ag-coated MNPs and then wide bioapplication [53]. During the particle’s synthesis, the SiO_2_ shell is coated onto the magnetic core to avoid its irregular aggregation, which can cause a weakened magnetic property and provide the outer noble metal shell with nucleation sites. Xu et al. [54] encapsulated Ag into yolk–shell-structured mesoporous SiO_2_ coated-Fe_3_O_4_ MNPs with bi-solvent and nanocoating technology using AgNO_3_ as a precursor to fabricate Fe_3_O_4_@Ag@mSiO_2_ NPS. The Fe_3_O_4_@SiO_2_@AgNPs-based SERS platform could achieve the reproducible and stable detection of biotargets.

The fabrication of a bimetallic shell is recognized as an effective route to achieve higher SERS activity [55,56,57,58]. Compared to a single-metal magnetic nanostructure, bimetallic and even trimetallic magnetic nanostructures also exhibit more outstanding SERS EM enhancement because of the synergistic effect of multimetals. Lin et al. [47] prepared the magnetic bimetallic substrate of Fe_3_O_4_@Ag@Au with a layer-by-layer coating strategy for the determination of cardiac troponin I (Figure 3B). This magnetic substrate with high signal enhancement uniformity exhibited a much higher Raman response than Fe_3_O_4_@Ag. A noble bimetallic alloy nanostructure usually possesses unique SERS performance [59], and magnetic SERS substrates based on bimetallic alloy nanostructures have been another research interest recently. Shen et al. [60] synthesized multifunctional magnetic Fe_3_O_4_@TiO_2_@Ag–Au through a galvanic replacement approach and applied it for catalysis and in situ SERS monitoring. Ag–Au bimetallic nanostructures significantly increased the effect of hot spots, offering stronger EM enhancement.

Furthermore, combining some materials with great adsorption capacity like MOFs and graphene-derived materials can prepare core–shell-structured magnetic nanocomposites like Fe_3_O_4_@Au/MOF [48] (Figure 3C) and Fe_3_O_4_@GO@Ag [49] (Figure 3D) as SERS substrates. These magnetic substrates could localize the analytes at the enhancing sites and generate versatile Raman enhancement. Sun et al. [61] prepared a magnetic MOF decorated with AuNPs as a SERS substrate (Fe_3_O_4_@UiO-66-NH_2_@Au). This magnetic substrate possessed the excellent adsorption capacity of UiO-66-NH_2_ for trace molecules and the strong SERS activity generated from abundant hot spots between adjacent AuNPs. Duan et al. [62] developed GO-wrapped Fe_3_O_4_@Au nanostructures as a sensitive magnetic substrate and separation tool for the detection of *Vibrio parahaemolyticus*. The SERS activity of this substrate was greatly improved by the combination of the EM enhancement of AuNPs and the chemical enhancement of GO. Prasiwi et al. [63] reported the chemical vapor deposition synthesis of the magnetic carbon nanofibers (CNFs) and investigated the SERS performance of CNFs using glycine as a model analyte. The CNFs exhibited great chemical enhancement due to the crystalline graphitic structure with accumulated electron transfer.

### 2.2. Magnetic Core–Satellite Nanoparticles

Plasmonic MNPs with satellite structures have been reported to exhibit great performance as SERS substrates due to their high density of multiple nano-satellites, which generate abundant hot spots at narrow interparticle gaps to provide significant EM enhancement [64]. The SERS activity of core–satellite MNPs is mainly related to the number and morphology of satellites and the interparticle distance. Pu et al. [65] prepared MNP@Au@MIL-100(Fe) magnetic substrate with a special core–satellite–shell structure through a seed-mediated growth approach and layer-by-layer strategy for the detection of malachite green in prawn. The generated high density of hot spots was attributing to the adjacent nano satellites of AuNPs, which provided strong EM enhancement. To achieve stronger EM enhancement than the MNPs with monolayer satellites, Zhao et al. [66] designed MNPs with a AuNP–AuNS (Au nanostar) bilayer-satellite nanostructure (BMPSNs). The architectonics of BMPSNs provided favorable conditions for the generation of multiple hot spots between adjacent AuNPs, AuNP–AuNS and the tips of AuNPs, which led to a lower limit of detection (LOD) for tobramycin as 0.44 fg/mL. In addition to varying the size and morphology of nano-satellites, controlling the interparticle distance of core–satellite MNPs with external magnetism is also an effective approach to tune the enhancement performance of the EM field. Han et al. [67] prepared satellite-structured Fe_3_O_4_@Au and modified it with an aptamer to construct a SERS aptasensor for the rapid detection of aflatoxin B1. The presence of the target would induce an immune reaction and then result in the formation of sandwich structures, which generated abundant ‘hot spots’ at the junction of the substrate and SERS tags (labeled AgNPs) to give EM enhancement. The second enhancement was induced by the magnetic trap effect, which resulted in the formation of hot spots between neighboring substrates. The LOD of the proposed method was as low as 0.0060 ng/mL due to the controllable hot spots and the external magnetic field.

The SERS activity of core–satellite MNPs is also related to the assembly methods of satellites. Thus, the interlayer has been commonly used in the fabrication of the stable and controllable satellites to increase the reproducibility. Mesoporous silica (mSiO_2_) has been a primary choice of interlayer materials thanks to its unique mesoporous structure, biocompatibility and optical transparency. Chen et al. [50] prepared Fe_3_O_4_@Ag/mSiO_2_/AuNPs (FASA) as a magnetic substrate to developed a label-free SERS-based method for the determination of methotrexate in serum. The mSiO_2_ interlayer was employed to anchor assembly AuNPs satellites on the surface and to concentrate the negatively charged methotrexate through the electrostatic interaction (Figure 3E). In addition, different from the above MNPs, a core–satellite nanostructure with plasmonic core and magnetic satellites has been also investigated. Achadu et al. [68] designed a core–satellite nanostructure by combining silver nanocubes and MNPs to achieve the dual-mode detection of norovirus. The developed synergistic dual-mode optical platform integrated SERS and fluorescence (FL) into a single probe for the rapid and ultrasensitive detection of norovirus.

Most of the current studies have focused on constructing core–satellite MNPs SERS substrates using Au NPs as assembled satellites due to their good biocompatibility that allowed the reliable modification of biorecognition factors onto the substrate surface to provide the selective detection of biotargets. Different from varying the morphology of satellites to tune the SERS performance of the substrate, manipulating the interparticle distance by magnetic field can allow the efficient generation of hot spots at the narrow gap of adjacent core–satellite MNPs to provide strong EM enhancement and further excellent SERS analysis sensitivity. The combination of mentioned approaches to construct multiple hot spots is also a promising means of providing more sensitive SERS methods for bioanalysis.

### 2.3. Non-Spherical Magnetic Nanoparticles

Non-spherical MNPs, which features a spherical magnetic core coated by different-shaped plasmonic materials, have obtained considerable attention as magnetic SERS substrates due to their strong EM enhancement originating from the branched structures. It was revealed that a flower-like and rough morphology benefited the Raman enhancement as compared to the smooth and spherical morphology [69]. As for the nanoflower-shaped core–shell MNPs, the branched structure of metal petal provided large numbers of hot spots, which significantly increased SERS activity. By incorporating binding materials between Fe_x_O_y_ and noble metal NPs, flower-like nanocomposites could be obtained. The Fe_3_O_4_@SiO_2_ nanospheres were usually modified by aluminate for the formation of nanoflower structures [70]. In a study performed by Huang et al. [51], γFe_2_O_3_@Au nanoflowers were synthesized through the iterative growth of a rough Au layer onto the surface of the γFe_2_O_3_ NPs. The SERS imaging of the flower-like γFe_2_O_3_@Au possessed ultrahigh sensitivity and could offer precise tumor localization and boundary (Figure 3F). Ding et al. [71] prepared the Fe_3_O_4_@Au@Ag nanoflowers via an in-situ reduction method. These Fe_3_O_4_@Au@Ag bimetallic nanoflowers with high ordered structure could provide a more reproducible and stronger Raman signal in SERS analysis.

The nanostar is another commonly used nanostructure in nanoscience. Like nanoflowers, star-shaped MNPs with plentiful spiky structures also possessed outstanding SERS activity attributed to the hot spots localized at the sharp tips and edges [72,73]. Quaresma et al. [74] developed star-shaped Fe_3_O_4_@Au MNP substrates for the SERS detection of protein. The Au seeds were synthesized on the surface of Fe_3_O_4_ NPs via the direct reduction of HAuCl_4_ by NaBH_4_ and subsequently grew up to be a completely star-shaped Au out layer. In this SERS strategy, the Raman signals were dramatically increased by the local amplification effect originating from the spiky morphology and the controllable aggregation of these nanostars induced by external magnetism. Reguera et al. [75] developed a consecutive seed-mediated approach to synthesize magnetic Janus-like nanostars, which started from Au seeds through dumbbell-like NPs to nanostars. Compared to previous Janus dumbbell-like NPs, this substrate could avoid the partial overlap between the Au lobes and FeO_x_ and a weak LSPR band. The star-shaped core–shell MNP substrates with more nanospikes usually offered abundant hot spots and magnetic responses during SERS analysis. Litti et al. [76] prepared the Janus-like Fe_3_O_4_/AuNSs as magnetic substrates in a microfluidic device to provide the SERS quantitative evaluation of erlotinib. The Fe_3_O_4_/AuNSs featured intrinsic hot spots at the spike structures, and these substrates could be retained at a specific position with a microfluidic channel by an external magnetic field to provide additional amplification of the Raman signal.

To date, the plasmonic MNP substrates with different shapes have been studied extensively, and the effect of shape and morphology on SERS activity has also been investigated systematically. The plasmonic MNP substrates with branched structures exhibited effective Raman enhancement due to the excellent plasmonic property. However, it is still a challenge to prepare non-spherical NPs with irregular shapes and high uniformity at the same time. Therefore, it would affect the efficient generation and ordered distribution of enhancing sites and result in disturbance for reproducible SERS signals during bioanalysis.

## 3. Functions of Magnetic SERS Substrates for Bioanalysis

### 3.1. Separation and Enrichment

**Figure 4:**
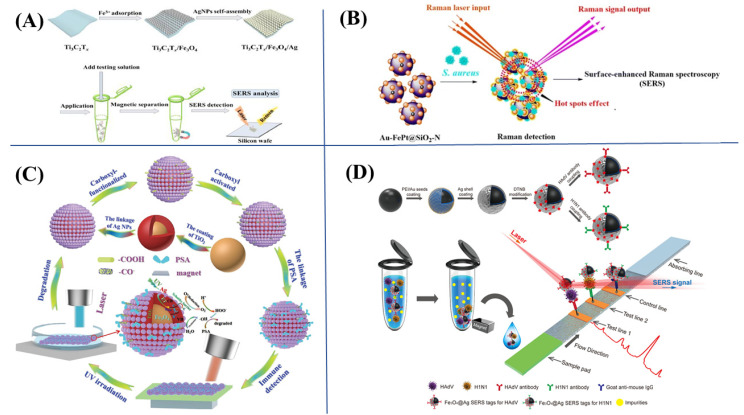
(**A**) Rapid SERS quantification for multiple trace sulfonamides in aquatic products based on magnetic Ti_3_C_2_T_x_/Fe_3_O_4_/Ag substrate. Reproduced with permission from Ref. [77]. Copyright 2022, Elsevier. (**B**) The mechanism of magnetic separation and SERS detection of *S. aureus* using Au–FePt@SiO_2_–N SERS platform. Reproduced with permission from Ref. [80]. Copyright 2015, Springer Nature. (**C**) The application Fe_3_O_4_@TiO_2_@Ag triplex core–shell MNPs for recyclable label-free SERS-based immunoassay of PSA. Reproduced with permission from Ref. [81]. Copyright 2020, Elsevier. (**D**) The magnetic SERS strip for detecting two respiratory viruses. Reproduced with permission from Ref. [82]. Copyright 2019, American Chemical Society.

The effective separation and enrichment of analytes in complex samples would benefit accurate SERS analysis. Recently, functionalized MNPs have been popular in SERS-based bioassays due to their excellent magnetic separation property, enrichment capacity and biocompatibility. Due to the induction of magnetic separation and enrichment function in SERS substrates all-in-one, the efficient concentration of biotargets followed by sensitive SERS detection can be achieved. Recently, our group [77] has prepared a magnetic Ti_3_C_2_T_x_/Fe_3_O_4_/Ag substrate to quantify trace antibiotics in fish and shrimp (Figure 4A). The Ti_3_C_2_T_x_/Fe_3_O_4_/Ag could simultaneously separate and enrich targets in aquatic products with magnetism to provide good analytical selectivity by SERS. The detection limits of phthalic sulfathiazole and silver sulfadiazine were low to 55.9 and 64.0 µg/kg, respectively, due to the synergy effect of the EM enhancement and chemical enhancement. Xiang et al. [78] described a SERS analysis method for trace cytidine in urine by Fe_3_O_4_/Au/Ag MNP substrates. The cytidine was absorbed on the surface of the MNP substrates dispersed in the sample solution, and then the mixtures were enriched in a capillary tube with an extra magnet for consequent SERS detection. By using the magnetic substrates, the proposed SERS method realized a dramatic improvement in analytical sensitivity that trace cytidine could be detected down to 1 nM without obvious matrix interference. Yu et al. [79] developed magnetic Fe_3_O_4_@GO@Ag substrates for the SERS detection of the illegal additive chloramphenicol (CAP) in diet honey coupling with SPME. The magnetic substrates were dispersed in the sample solution to adsorb the analytes on the surface. Then, SPME was employed to enrich Fe_3_O_4_@GO@Ag and CAP on the needle tip for further SERS detection. The simultaneous extraction, enrichment and SERS detection was successfully achieved by the use of these MNP substrates with an excellent LOD of 1.0 × 10^−10^ mol/L.

Apart from the analysis of specific chemical substances in biological samples, the analysis of microbial contaminants by SERS has also aroused much attention recently. However, most of the existing methods required a cumbersome sample preparation procedure and a lengthy assay time. The introduction of MNP SERS substrates could integrate the magnetic enrichment–separation and in situ SERS detection, which greatly benefited the efficient and accurate SERS analysis of real biosamples. Wang et al. [83] firstly reported a SERS-based method for the rapid, sensitive and label-free detection of bacteria pathogens utilizing magnetic substrates of Fe_3_O_4_@Au@PEI MNPs. In this strategy, the Fe_3_O_4_@Au@PEI was employed to capture target bacteria through electrostatic interactions, and the SERS signal was further enhanced due to the conjunction of Fe_3_O_4_@Au@PEI MNPs and Au@Ag. This method offered a selective and accurate analysis strategy for bacteria. Similarly, Hardiansyah et al. [80] prepared the magnetic FePt@SiO_2_–Au substrate to provide the rapid SERS detection of small biomolecule and identification of *Staphylococcus aureus* (Figure 4B). In this work, amine-functionalized FePt@SiO_2_–Au with a positive charge could capture negatively charged *Staphylococcus aureus*. The separation process was easily operated by an extra magnetic field for further SERS detection. The employment of FePt@SiO_2_–Au substrate achieved the simultaneous magnetic separation and enrichment of *Staphylococcus*. Yang et al. [39] prepared magnetic Fe_3_O_4_–Au/RGO nanosheets and used them as both SERS substrate and bacteria capture materials. The Fe_3_O_4_–Au/RGO exhibited outstanding capturing efficiency toward *S. Aureus*, at 67 ± 5%, and could be employed to identify *S. aureus* down to 1 × 10^4^ CFU. Juang et al. [84] also developed a magnetic in situ capturing and SERS detection method for bacteria using Fe_3_O_4_@AuNPs@nanoscale silicate platelet (NSP) nanosheets as substrate. The prepared magnetic substrate was fabricated by immobilizing Fe_3_O_4_@AuNPs on NSP nanosheet, enabling the efficient capture of bacteria and amplification of the Raman signal.

Functionalized MNP substrates usually enable effective separation and enrichment after a successful capture of analytes by an extra magnetic field, which can effectively avoid the interference effect from other biomolecules existing in the sample. The property of magnetic composites loaded with SERS-active NPs will be diverse due to their different structures and compositions, which will greatly influence the separation–enrichment capacity of the consequently synthesized MNP substrates. Therefore, combining various nanomaterials with excellent functional properties, such as high surface-area-to-volume ratios, selective adsorption and chemical SERS enhancement can provide more promising potential to achieve the selective and sensitive analysis of more trace biotargets by SERS in complex biological samples.

### 3.2. Recognition

In order to achieve the selective SERS detection of biotargets in complex samples, recognition molecules, such as antibodies, aptamers, peptides and other small molecules, have been utilized to modify the MNP-based substrates for specifically capturing the analytes. On the other hand, such functional groups, including carboxyl (-COOH), amino (-NH_2_), hydroxyl (-OH) and sulfhydryl (-SH), are frequently introduced to enhance the recognition capability of MNP substrates. Au-coated MNPs are widely used for the surface modification of SERS substrates. Tamer and co-workers et al. [85] synthesized Au-coated MNP substrates with recognition surface for the detection of *Escherichia coli*. The Fe_3_O_4_–Au MNPs were modified by two component self-assembled monolayers to form a recognition surface and provide the effective capture of bacteria. Shown in Figure 4C, Du et al. [81] designed a novel multifunctional Fe_3_O_4_@TiO_2_@Ag magnetic substrate to construct a label-free SERS immunoassay for the determination of prostate-specific antigen (PSA). After PSA was linked onto the surface of carboxyl-functionalized Fe_3_O_4_@TiO_2_@Ag substrates, the SERS response was triggered. Thereafter, successful photocatalytic degradation was introduced to achieve the recyclable detection of PSA with an LOD of 16.25 pg/mL. Molecularly imprinted polymers (MIPs) have been also widely used to prepare MNP substrates with specific recognition capability due to the molecular-size selection effect. Zhang et al. [86] prepared an Fe_3_O_4_@Au@*β*-cyclodextrin (*β*-CD)–MIP substrate for the ultrasensitive SERS detection of transferrin (TRF). Herein, *β*-CD was employed as a functional monomer to synthesize MIPs with hydroxyl groups and hydrophobic cavities. Thus, the synthesized Fe_3_O_4_@Au@*β*-CD–MIP substrate can provide both the selective enrichment of TRF via the noncovalent interaction and the excellent SERS activity for consequent bioanalysis.

Compared with chemically modified functional groups, introducing corresponding antibodies into the preparation of MNP SERS substrates usually offers higher specificity/selectivity due to their special interactions with specific antigens. Currently, MNPs modified with antibodies are usually employed as high-recognition substrates to develop various SERS-based sandwich immunoassays. Shin et al. [87] synthesized the Fe_3_O_4_@SiO_2_@Au MNP substrate for immunoglobulin G (IgG) detection using SERS-based immunoassay. Different antibodies of mouse IgG and human IgG were deposited onto the surface of Fe_3_O_4_@SiO_2_@Au MNPs to selectively capture both mouse IgG and human IgG, respectively. The developed immunoassay could achieve the detection of these proteins at extremely low concentrations (~800 ag/mL for mouse IgG and ~5 fg/mL for human IgG). Du et al. [88] prepared a magnetic immune probe of Fe_3_O_4_@TiO_2_@Au for developing a recyclable SERS-based immunoassay for the determination of PSA. This immunoassay was feasible after even six cycles of analysis.

Like antigen–antibody-specific interaction, aptamers can also specifically bind to biotargets. Since aptamers have ease of modification and synthesis and possess good stability, this makes them a more effective choice than antibodies to prepare high-recognition magnetic substrates for SERS bioassays. Zhao et al. [89] developed an aptamer-modified Fe_3_O_4_@Au nanocomposite biosensor for the SERS detection of *Staphylococcus aureus*. The prepared MNP substrates showed outstanding ability for capturing *Staphylococcus aureus* (68%) after surface modification with nucleic acid aptamer. Wang et al. [90] prepared magnetic MnFe_2_O_4_@Ag substrates and conjugated them with the aptamer of *Staphylococcus aureus* to effectively and specifically capture and enrich the target. Once target bacteria existed in the system, the special interaction between the aptamer and target occurred and resulted in the formation of a substrate–target–SERS-tag sandwich structure for the consequent SERS quantification of *Staphylococcus aureus.*

In addition, the MNP substrates conjugated with multiple antibodies or aptamers as the substrate could achieve the multiplex and simultaneous analysis of different biological targets by SERS. Wang et al. [82] synthesized the multiple-target-antibodies-modified Fe_3_O_4_@Ag MNPs as magnetic SERS tags for the construction of a magnetic SERS strip to simultaneously detect respiratory viruses (Figure 4D). The synthesized magnetic nanotags could specifically recognize and further enrich the target viruses by magnetism. Li et al. [91] established an ultrasensitive SERS-based immunochromatographic method to sensitively detect various foodborne bacteria at the same time by employing Mag@Au–BSA nanotags as the substrate. The proposed method could minimally and simultaneously detect 12 cells/mL of *Salmonella typhimurium* and 9 cells/mL of *Staphylococcus aureus* in 30 min.

Generally, bare MNPs have weak recognition ability towards biotargets, peculiarly in complex samples. Thus, many surface-modification strategies have been developed to address the issue by causing more adsorption interactions, such as antigen–antibody-specific interaction, π–π stacking, electrostatic interaction and hydrogen bonding. Through surface modification, the recognition ability of MNP substrates has been significantly improved during the SERS analysis of complex biosamples. The combination of MNPs and functional material that can provide covalent interactions with the analytes will further provide more selective and stable magnetic SERS substrates for biological analysis.

### 3.3. SERS Tags

The SERS detection of the mentioned label-free method mainly relies on distinguishing fingerprint peaks of the analyte, which could be disturbed by the presence of unexpected peaks resulted from matrix interference. Therefore, Raman reporter-molecule-labeled MNPs have been employed as both substrates and SERS tags to provide accurate and quantitative results of biotargets in complex samples. Jun et al. [92] prepared AgNP-embedded MNPs (M-SERS dots) and used them as SERS nanoprobes for SERS application. The M-SERS dots could specifically recognize the target cells through antigen–antibody interactions. After magnetic separation, the targeted cancer cells exhibited significant SERS signals generated from the M-SERS dots. Qiu et al. [93] proposed a SERS immunoassay to detect cancer cells at low-level concentrations using 4-ATP-labeled Fe_3_O_4_–Au as SERS tags. In the presence of carcinoembryonic antigen (CEA), both the SERS tags and anti-AuNPs bound to the target through the antigen–antibody interaction, and the SERS-tags–CEA–AuNPs sandwich structure formed, which significantly enhanced the Raman signal of the 4-ATP localized at SERS tags. Based on the enhanced Raman signal, this immunoassay could be used to detect CEA with an LOD of 10 cells/mL. Compared with the construction of hot spots, the fine-tuning distance between Raman reporters and SERS substrates through a hairpin-structure probe is more facile and easier to obtain enhanced Raman signals for the quantification of the analyte. In the study by Chai’s group [94], an “off–on” SERS bioassay was developed to detect microRNA using hairpin-DNA-labeled magnetic Co@C/PEI/Ag as the SERS tag. A reporter of Cy-5 was localized at the end of hairpin-DNA. This SERS strategy achieved the change from “off” to “on” by tuning the distance between Cy5 and Co@C/PEI/Ag through DNA hybridization. This SERS platform could achieve the quantification of MicroRNA 155 in a very low content range from 100 aM to 100 pM. Conversely, a SERS platform based on reverse attenuation was successfully developed to identify the tetracycline resistance gene *tetA* using core–satellite-structured Fe_3_O_4_@SiO_2_–Au as a SERS tag by Lu et al. [95] The target DNA sequence would lead to a structural change in the hairpin-structured-probe, push the Cy-5 Raman reporter away from the Au satellites and result in a significant decrease in the SERS signal that originated from Cy-5. The LOD of *tetA* could be achieved as 25 copies/μL. Similarly, Bedford et al. [96] reported that the Raman label bound to a hairpin probe would stay away from Fe_3_O_4_@Au upon complementary DNA hybridization, which could lead to a significant decrease in the Raman signal for the quantification of target DNA sequence.

In summary, the employment of magnetic SERS tags consisting of a magnetic substrate and Raman reporter exhibited great potential for the quantitative measurement of DNA by SERS. Currently, most of the reported label-based SERS methods used the individual SERS signal generated from the Raman reporter to quantify the analyte. It is expected to be a promising trend in this field that more reliable SERS tags based on the ratio of SERS signals originating from multiple Raman reporters will be developed for the accurate determination of biomarkers.

## 4. Applications of Magnetic SERS Substrates for Bioanalysis

As illustrated above, SERS is a powerful technology for biotarget detection, and the substrate plays an important role in SERS performance. Virtually no magnetic background in biological environment can lead to a highly sensitive SERS analysis.

The MNP-based substrates possess facile enrichment and separation capacity, which can reduce the matrix interference effect and simplify the pretreatment process to ensure the efficiency of SERS detection. The recognition elements loaded on the surface of MNP-based substrates enable the selective SERS detection. In this section, we will review the typical applications of SERS bioanalysis through the use of MNP-based substrates recently. Some typical applications of magnetic substrates for SERS analysis of biotargets are summarized in Table 1. As shown in Table 1**,** amino acids and protein, DNA and RNA sequences and tumor biomarkers are the main biotargets attracting the researchers’ interest in this field.

### 4.1. Amino Acids, Protein and Their Derivatives

In the biomolecules of the human body, amino acids are essential substances to construct cells and repair injured tissues. The disorder states of amino acids may lead to very serious health problems. Therefore, researchers have developed various functionalized MNP-based SERS methods to detect different kinds of amino acids. Wu et al. [109] synthesized Ni@CNFs@Au NPs as magnetic substrates for the SERS analysis of phenylalanine that was linked with the liver disease. The Ni@CNFs@Au substrate could greatly increase the Raman signal of phenylalanine due to its high density of hot spots, and an ultrasensitive LOD of 1 × 10^–11^ M was achieved finally. Zhou et al. [110] combined carboxyl-modified MNPs and Au@ATP@Ag to fabricate a sandwich nanosensor for the determination of histamine. The LOD of the proposed method in fish samples and RAW264.7 cell lysates were 20 µg/kg and 2 µM, respectively.

**Figure 5:**
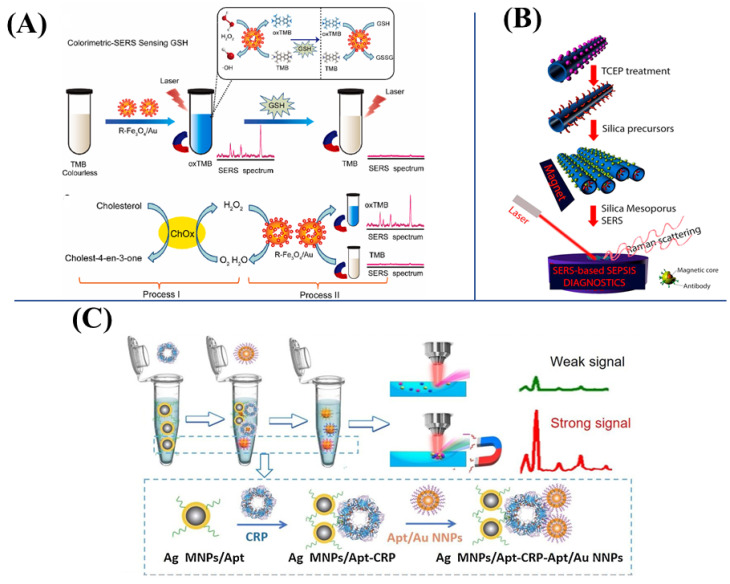
(**A**) The SERS-FL dual-mode detection of GSH and cholesterol based on R–Fe_3_O_4_/Au. Reproduced with permission from Ref. [112]. Copyright 2022, Elsevier. (**B**) The SERS-based triplex assay using Au-coated magnetic NCs for sepsis diagnostics. Reproduced with permission from Ref. [120]. Copyright 2016, Elsevier. (**C**) The illustration of protein detection via Ag MNPs–CRP–Au NNPs “sandwich” structure by SERS. Reproduced with permission from Ref. [121]. Copyright 2021, Elsevier.

Amino acid derivatives closely related with amino acids are a kind of crucial biomarkers in biomedical and clinical science. For example, glutathione (GSH) as a typical amino acid derivative plays an important role in controlling biochemical reactions associated with human health. In order to monitor the content of GSH in cells, a series of magnetic SERS biosensors have been developed. Saha et al. [111] developed a “turn off” strategy of the SERS signal for the determination of GSH and oxidized GSH using γ-Fe_2_O_3_–Au as the magnetic substrate. This strategy relied on the GSH-introduced replacement of a Raman probe localized on the surface of the magnetic substrate. The SERS signal was linearly correlated in a GSH concentration range of 1 pM~10 μM, and the approach could be applied to detect low-level GSH in a single cell. Ouyang et al. [112] reported a magnetic SERS method via the competed adsorption between crystal violet and GSH on Fe_3_O_4_/Ag MNPs. Raman-insensitive GSH could partly replace the Raman probe immobilized on the surface of Fe_3_O_4_/Ag MNPs, resulting in a strong decrease in the SERS signal that was utilized to determine the GSH. The employment of Fe_3_O_4_/Ag with good stability guaranteed the reproducibility of the proposed method, and the LOD was determined to be 40 nmol/L. In order to obtain more reliable and robust performance in the detection of GSH, Huang et al. [113] developed a SERS/colorimetric dual-mode sensing method using magnetic R–Fe_3_O_4_/Au substrates with peroxidase-like property (Figure 5A). The R–Fe_3_O_4_/Au with the peroxidase-like property could effectively catalyze the TMB–H_2_O_2_ reaction and generate oxidized products (ox-TMB) as Raman probe and concomitantly served as SERS substrates to detect the Raman signal of ox-TMB. GSH in the system could capture the free radicals generated by H_2_O_2_, which led to the decreasing production of ox-TMB and further resulted in the changing color of the reaction solution and the decrease in the SERS signal. Based on the redox reaction, the SERS/colorimetric sensing system achieved an LOD of GSH as low as 0.10 μM.

Proteins that usually comprise one or more long chains of amino acid residues are essential substances of the human body. Proteinases are the most common proteins that can catalyze various biochemical reactions and play an important role in biological metabolism. Many magnetic SERS substrates were prepared for detecting proteins and proteinases. Yap et al. [114] developed microfluidic SERS immunoassay-based magnetic Fe_3_O_4_@AuNPs@Ag bifunctional particles for the determination of Ig G. The proposed microfluidic system could detect the rabbit IgG down to 1 pg/mL in 80 min using magnetic Fe_3_O_4_@AuNPs@Ag as SERS immunosubstrate to promote micromixing. Zhao et al. [115] developed a magnetic SERS-based immunoassay to detect metalloproteinases 9 (MMP-9) using the corresponding antibody-conjugated magnetic beads as capture probes. In the optimal conditions, the LOD of the proposed immunoassay was 1 pg/mL, much lower than the minimum detectable concentration of the commercial assay kit, which was 50 pg/mL. Wei et al. [116] proposed a SERS-based platform to provide O-GlcNAc transferase (OGT) determination by using a protease protection strategy. In the assay, the SERS tags and rod-shaped MNPs could be linked to the two ends of the designed peptide to form a sandwich-structure SERS substrate for sensing OGT. The designed peptide containing glycosylation and protease sites allowed the occurrence of peptide glycosylation, which avoided the cleavage by proteinase K and ensured the stable connection between SERS tags and rod-shaped MNPs that contributed a high SERS signal for the quantification of OGT. The developed strategy achieved an LOD of OGT that was 0.1 nmol/L. In addition, combing through various designed peptide substrates, the method could exhibit the wide universality of SERS performances toward other glycosyltransferases. Cardiac troponin I (cTnI) has been recognized as a protein biomarker of acute myocardial infarction. Lin et al. [117] developed a coral-like magnetic substrate of Fe_3_O_4_@PEI/AgNC for the highly sensitive determination of cTnI. The Fe_3_O_4_@PEI/AgNC was modified by aptamer to capture cTnI. The LOD of the proposed SERS method was 0.23 pg/mL. Similarly, Alves et al. [118] fabricated an aptasensor for the SERS detection of cTnI using a flower-like magnetic substrate of Fe_3_O_4_@SiO_2_@Ag. The branched structure combined with magnetic aggregation resulted in the efficient generation of hot spots between neighboring magnetic substrates to amplify the Raman signal. The LOD of this method was confirmed to be 10 ng/mL. Tamer’s group [119] presented a SERS quantification method for recombinant erythropoietin (rEPO) in urine using the core–shell-structured magnetic AuNPs of Fe_3_O_4_@Au as the extraction tool. The LOD of this method was lower than 0.1 pg/mL due to the excellent extractive performance of the Fe_3_O_4_@Au. When the sandwich immunocomplex formed, the SERS signal of Raman reporter molecules could be significantly amplified due to LSPR between magnetic supporting materials and SERS tags and then were used for SERS quantification. In order to detect the biotargets associated with sepsis, Nguyen et al. [120] developed Au-coated magnetic nanostars (Au-MNSs) as substrates to construct a SERS-based triplex assay (Figure 5B). The proposed method could simultaneously detect three sepsis-specific biotargets with LODs of 27–103 pM. Hu et al. [121] designed a SERS platform using Fe_3_O_4_@Ag as magnetic capture substrate for the ultrasensitive detection of CRP (Figure 5C). This strategy exhibited high sensitivity toward CRP with an LOD of 1.14 pg/mL and could provide the special and accurate quantification of CRP in complex samples containing other proteins.

### 4.2. DNA and RNA Sequences

DNA and RNA are the two main kinds of nucleic acids that have been considered crucial biopolymers and macromolecules that can store, encode and express genetic information and are associated with different kinds of cellular events and diseases. The rapid and accurate quantification of specific DNA and RNA sequences is important for diseases diagnosis, so various efficient SERS biosensors assisted by magnetic substrates for the bioanalysis of DNA and RNA sequences have been developed. Liang et al. [122] employed Ag@SiO_2_ as Raman tags and SiO_2_-coated MNPs modified with -NH_2_ as a capture probe to detect DNA sequences related to HIV. Ngo et al. [123] developed a SERS method based on a sandwich hybridization strategy to detect malaria-related DNA and the discrimination of single-nucleotide polymorphism using MNP substrates. The LOD of the proposed method was approximately 100 attomoles for *Plasmodium falciparum* target DNA. Strelau et al. [124] used streptavidin-modified MNPs for the enrichment and purification of the biotargets and achieved the sequence-specific detection of DNA by SERS. The MNPs were used as a separation tool for the further purification and enrichment of the target DNA strands. After the hybridization procedure was completed in solution, a dye-modified, short designed single-stranded (ssDNA) served as the SERS label to reflect the existence of the target DNA sequence. The real-time monitoring of DNA-involved reactions is also essential. Lin et al. [125] synthesized bifunctional MNPs as bifunctional nanoprobes to achieve SERS monitoring and provide magnetic intervention to biochemical reactions of target DNA. With the magnetic intervention, MnZn ferrite NPs (MZF/Au) could be linked with Raman-probe-labeled AuNPs through the direct recognition of two DNA strands, resulting in the generation of interparticle hot spots and the enhancement of the SERS signal. The bifunctional MNPs exhibited great potential to integrate biomolecular recognition and intervention. Based on the hairpin-structured strategy, Wu et al. [126] also developed a sensing system of *Bacillus thuringiensis special gene* using SERS. In the presence of the target, the biotin would be exposed due to the hybridization between target and hairpin, and then suspended rod-shape Au NPs were captured by streptavidin-modified MNPs, which led to the decrease in Raman intensity that was used to quantify target genes. This strategy exhibited high sensitivity toward target genes with an LOD of 0.14 pmol/L.

MicroRNAs (miRNAs) usually containing 19–23 nucleotides are single-stranded noncoding RNAs. miRNAs can regulate the cleavage and translational repression of mRNA and are also regarded as potential biomarkers for the diagnosis of diseases. Yang et al. [127] reported a sandwich strategy based on Fe_3_O_4_@Au branched-type MNPs to detect miRNA-21 by SERS. The tetrahedral DNA was immobilized onto the surface of the branched Au-coated MNPs to construct capture probes for sample separation and target preconcentration. The established method provided high sensitivity and specific selectivity with an LOD of 623 amol/L. However, the sensitive and accurate quantification of miRNAs by SERS is still a challenge due to their small size, sequence homology and low abundance in real biosamples. Therefore, some signal amplification strategies have been exploited to construct highly sensitive analytical methods by SERS. For example, by coupling with a duplex-specific nuclease (DSN)-based signal amplification strategy, a label-free SERS method was established for the quantitative analysis of miRNA-21 by Yao et al. [128]. Magnetic beads with capture DNA were used for the hybridization of the target miRNA-21. The DSN was employed to cleave the DNA–RNA heteroduplexes to release nucleotide fragments of capture DNA that possessed abundant phosphate backbones, resulting in the transformation and amplification of miRNA-21. Similarly, Chai and co-workers [94] combined the SERS technique and a exponential rolling circle amplification strategy to construct a SERS-based platform for miRNA 155 detection. The triggered DNA obtained from the P-ERCA process could be complemented with placeholder DNA to recover the hairpin structure, which made the Raman label reclosed to the Co@C/PEI/Ag and generate a strong SERS response. Based on this “off–on” mode of SERS response, this SERS platform could detect 100 amol/L~100 nmol/L miRNA 155, and the LOD was found to be 70.2 amol/L.

### 4.3. Cancer Cells and Related Tumor Biomarkers

Cancer, in contrast to benign tumors, is a group of diseases involving abnormal cell growth and has been one of the most life-threating diseases. A late disease diagnosis has been acknowledged to be the main factor resulting in huge numbers of deaths related to cancer. Therefore, many strategies aiming for cancer-cell sensing have been developed to provide early diagnosis and the effective treatment of cancer. Jun et al. [92] prepared magnetic SERS dots based on Fe_3_O_4_@SiO_2_@Ag nanobeads combined with an antibody for the separation and detection of breast-cancer cells (SKBR3) and floating leukemia cells (SP2/O). After efficient magnetic separation, the targeted cancer cells exhibited strong SERS signals by magnetic SERS dots. The formation of circulating tumor cells (CTCs) has been considered from the metastasis of cancer cells into the vasculature. The accurate determination of the concentration of CTCs could reflect the condition of cancer, which is of great significance for prognosis. Xue et al. [129] designed an improved MNP-based substrate and applied it to provide the sensitive and quantitative analysis of CTCs by SERS. The superparamagnetic iron oxide NPs (SPION) was coated by PEI and a Au shell, and then the particles obtained were modified with the Raman reporter molecule of MBA and folic-acid-conjugated reduced BSA to form magnetic substrates for the enrichment and magnetic separation of CTCs and further SERS testing. The experiments exhibited excellent sensitivity toward Hela cells with an LOD of 1 cell/mL.

**Figure 6:**
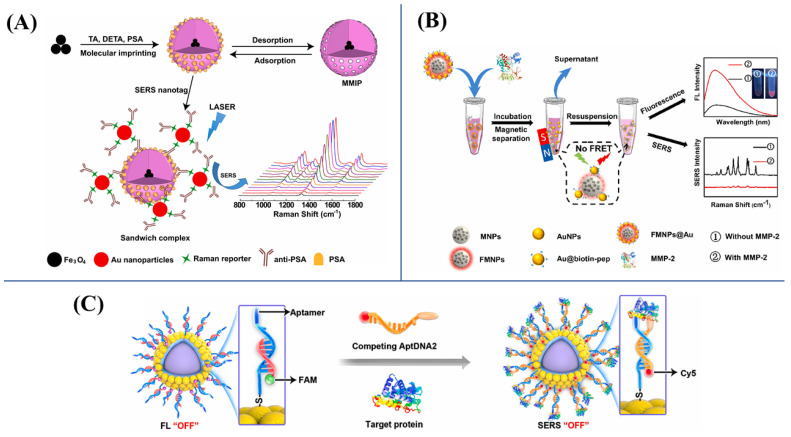
(**A**) Construction of SERS-based platform for PSA detection using MMIPs. Reproduced with permission from Ref. [131]. Copyright 2021, Elsevier. (**B**) Sensing principle of the FMNS@Au dual-mode nanosensor for MMP-2. Reproduced with permission from Ref. [134]. Copyright 2022, Elsevier. (**C**) The DNA strand displacement strategy for SERS-FL dual-mode analysis of vascular endothelial growth factor. Reproduced with permission from Ref. [135]. Copyright 2022, Elsevier.

Apart from cancer cells, the sensitive detection of tumor biomarkers will also provide an accurate criterion for the early diagnosis and medical treatment of cancer. Currently, many SERS bioassays using MNP substrates have been constructed for the sensitive and selective analysis of tumor biomarkers. Zong et al. [130] developed a SERS immunoassay to realize the facile determination of tumor-derived exosomes. Magnetic Fe_3_O_4_@SiO_2_ beads combined with Au@Ag nanorods were synthesized as the substrates, and the aptamer was linked to their surface for the specific recognition of target exosomes. In the system, the strong SERS signal generated due to the formation of the sandwich immunocomplex, which could be used for the sensitive quantification of exosomes secreted by SKBR3 cells with an LOD of 268 amol/L. Tamer’s group [131] designed a SERS biosensor to detect PSA by using magnetic molecularly imprinted polymers (MMIPs), as shown in Figure 6A. Owing to the antigen–antibody-specific interaction, the prepared MMIPs labeled with Au NPs were modified by anti-PSA and a Raman reporter to form a sandwich complex, which generated hot spots at the place where Raman reporters localized. Utilizing the amplification of the SERS signal, PSA could be detected with a low LOD of 0.9 pg/mL and an LOQ of 3.2 pg/mL, respectively. CEA is a typical biomarker for colorectal cancer. There have been several magnetic SERS immunosensors developed for the sensitive detection of trace CEA. Medetalibeyoglu et al. [132] prepared a sandwich SERS immunosensor based on magnetic Fe_3_O_4_@AuNPs/d-Ti_3_C_2_T_X_ as the substrate and MoS_2_@Au NPs as the SERS tag for CEA detection. In the presence of CEA, the immunoreaction dragged SERS substrates and SERS tags into proximity, leading to EM enhancement and further signal amplification. This sandwich immunosensor could minimally detect 0.033 pg/mL CEA. Similarly, Song [133] et al. proposed a SERS immunoassay to simultaneously detect trace CEA and neuron-specific enolase (NSE) in human serum using magnetic substrate. The low LODs of CEA and NSE were 1.48 and 2.04 pg/mL, respectively, which suggested the excellent sensitivity of the proposed magnetic SERS bioassays. Liu et al. [134] prepared core–satellite fluorescent–magnetic nanospheres (FMNS) @Au for the real-time detection of matrix metalloproteinase 2 (MMP-2) activity. The presence of MMP-2 could result in the quenching SERS signal and the recovery of fluorescence emission, and the dramatic change of dual-mode signals could be utilized to quantify trace MMP-2. The proposed dual-mode platform could be applied to minimally detect 1.17 ng/mL MMP-2 (Figure 6B). Our group [135] designed a DNA strand displacement strategy to develop a SERS-FL dual-mode method for SERS quantification and the FL imaging of vascular endothelial growth factor using magnetic dual-mode nanoprobes (Figure 6C). The proposed SERS-FL dual-mode method exhibited high sensitivity toward VEGF with an LOD of 2.3 pg/mL in SERS-quantification mode and could also provide the distribution information of trace VEGF in living tumor cells in FL-imaging mode.

### 4.4. Other Biomolecules

Other biomolecules, such as hormones, metabolites and antibiotics, also play important roles in various physiological processes. Hormones are a class of signaling molecules, or so-called chemical messengers, that can affect many processes to regulate physiology and behavior. For example, it is a natural response for the human body to produce cortisol in stressful situations. Cortisol, also called the stress hormone, is regarded as the principal stress biomarker. Villa et al. [136] developed a cortisol biosensor based on a magnetic SERS immunoassay strategy. They prepared the magnetic bead substrates modified by the cortisol antibody that could capture the cortisol, selectively bind to the SERS tags and further generate a SERS signal to quantify the target. Testosterone is an important steroid hormone produced by both males and females. Liu et al. [137] developed a competitive SERS immunoassay for the analysis of trace free testosterone. Magnetic beads were labeled with an antibody to capture testosterone, and the specific antigen was immobilized on the surface of SERS tags. The developed magnetic SERS immunoassay possessed great potential application for rapid disease diagnosis through analyzing trace levels of free testosterone, which could be as low as the fg level. The competitive adsorption occurred in the presence of testosterone, leading to a low SERS signal. Human chorionic gonadotropin (hCG), related to testosterone, can stimulate testosterone production and affect the size and performance of muscles. Tamer’s group [138] designed a SERS immunoassay based on a capillary-driven microfluidic chip for the determination of hCG through the use of magnetic MOF substrates. This proposed magnetic SERS immunoassay demonstrated good selectivity for trace hCG in urine and achieved an LOD down to 0.61 IU/L.

Bacteria and their secreta are essential biomarkers for environmental, agricultural and food science. Much effort has gone to develop fast analytical methods for the typical bacteria and their secreta, and the MNP-substrates-based SERS method is one of the promising choices. Weng et al. [139] developed an all-in-one magnetic nanosensor for the rapid analysis of *Escherichia coli*. The target cells could be first captured by the nanosensors and magnetically enriched within 15 min. This magnetic nanosensor was sensitive, reproducible, low-cost and easy for use, demonstrating good practical potential. Chattopadhyay et al. [140] designed a SERS immunosensor using polymeric MNPs as an effective capture probe and magnetic separator for the detection of *S. typhimurium* coupling with two specific Raman reporters. Under optimal conditions, this magnetic SERS immunosensing method achieved an LOD down to 10 cells/L and showed strong potential for in situ monitoring the contamination in food samples.

## 5. Conclusions and Perspective

SERS has been an attractive and powerful technology for bioassays due to the fact that SERS can provide rapid and non-destructive detection with water-free interference. The employment of MNP-based substrates for SERS bioassays can not only achieve simultaneous separation, enrichment and SERS detection to improve the efficiency of SERS analysis but also allow the introduction of many kinds of recognition factors due to their good biocompatibility, which greatly improved the analytical selectivity and accuracy of the established SERS methods for complex biological samples. Moreover, the MNP-based substrates can be easily functionalized to exhibit specific performance, including the separation and enrichment tool, SERS tags and capture probes, along with their excellent Raman enhancement performance, which broadens their application range in biological analysis.

Although MNP-based SERS substrates have been widely applied in the field of bioanalysis, there are still some limitations to be addressed in future studies. (1) Most of the proposed MNP-based SERS methods required MNP-based substrates with high uniformity, reproducibility and chemical stability. However, it is difficult to obtain these functionalized MNPs via simple synthesis, which ca not meet the requirement for high efficiency in mass production and application. Thus, more efforts should be put on the development of advanced nano-synthesis approaches. (2) It is still difficult to provide an accurate quantitative result of trace biotargets in biological samples. Label-free MNP-substrate-based SERS methods rely on the distinguishing ‘fingerprint’ peaks of the analytes to reflect their actual existence. However, the environmental interference may result in multiple spectral peaks and would interfere with the qualitative and quantitative information of the analytes. In addition, these methods are not suitable for some biotargets with weak or no Raman signal response. To improve their practicality, they should be combined with other techniques, such as the derivation strategy, which can transfer these biotargets to derived products with high SERS activity. On the other hand, most of the proposed labeled MNP-based SERS methods determined the analyte using the individual SERS signal generated from the Raman reporter, which can not reflect the real existence of the analyte when competitive adsorption exists in the system. The employment of ratiometric SERS methods is usually necessary to provide a more reliable quantitative result. In addition, combining SERS with other spectra techniques to construct a multiple-mode method is also beneficial to the accurate quantification of biotargets. (3) Clinical diagnosis depends on the simultaneous determination of multiple substances in biological samples. Most of the reported MNP-substrate-based SERS methods focused on the analysis of a single biotarget in a limited number of samples, which would limit their analysis throughput and applicability in clinical diagnosis. It is hoped that the combination of MNP-substrate-based SERS methods and emerging techniques, such as multichannel microfluidics and layer flow immunoassays, would improve the analytical throughput based on the realization of the simultaneous determination of multiple targets. Overall, it is highly expected that, with great research advances in MNP-based substrates, SERS technology can grow into a futuristic choice for practical bioanalysis.

## Figures and Tables

**Table 1:** Typical application of magnetic substrates for SERS bioanalysis.

Category	Biotargets	Magnetic Substrates	LOD	Ref.
Amino acid and protein	Uric acid	Ag/ZnO/Fe_3_O_4_	365 nmol/L	[97]
	Glycated hemoglobin	Ag-coated magnetic polymethacrylate microspheres	50 ng/L	[98]
	Human carboxylesterase 1	Fe_3_O_4_@SiO_2_@Ag	0.1 ng/L	[99]
DNA and RNA sequences	ssDNA associated with BRAF V600E mutation	MNP@SiO_2_@Au	5.15 × 10^−11^ mol/L	[100]
	*C. krusei* and *C. albicans* target DNA	Ag@MNP	20 fmol/L	[101]
Tumor biomarker	CEA	Raspberry-like Fe_3_O_4_@Au	1.43 pg/mL	[102]
Bacteria and secretions	Deoxynivalenol	Complementary DNA modified-Fe_3_O_4_@Au	0.032 pg /mL	[103]
	Zearalenone	Fe_3_O_4_@Au	0.001 ng/mL	[104]
	*E. coli*, *L. mono* and *S. typhi*	Aptamer modified-Fe_3_O_4_@Au	10 cells/mL (*E. coli*), 10 cells/mL (*L. mono*) and 25 cells/mL (*S. typhi*)	[105]
	*E. coli*, *S. aureus* and *Salmonella*	Magnetic Au@Ag	20 cells/mL (*E. coli*), 13 cells/mL (*S. aureus*) and 19 cells/mL (*Salmonella*)	[106]
Hormones and antibiotics	Luteinizing hormone	Antibody modified-Fe_3_O_4_@AuNP	0.036 IU/L	[107]
	CAP	CAP antibody modified-MNPs	1.0 pg/mL	[108]

## Data Availability

The data presented in this study are available on request from the corresponding author.
